# Application of new media-based home nursing intervention for stroke-related depression patients: a retrospective cohort study

**DOI:** 10.3389/fpubh.2026.1775705

**Published:** 2026-03-09

**Authors:** Xuhui Huang, Xiaona Tang, Xiaoli Yang, Xiaoyang Wang, Lan Song, Meizhen Lin

**Affiliations:** 1 Traditional Therapy Center, Shenzhen Bao'an Traditional Chinese Medicine Hospital Group, Shenzhen, China; 2 Nursing Department, Shenzhen Bao'an Traditional Chinese Medicine Hospital Group, Shenzhen, China; 3 Classic Ward Geriatrics Department, Shenzhen Bao'an Traditional Chinese Medicine Hospital Group, Shenzhen, China; 4 Acupuncture and Moxibustion Rheumatology Department, Shenzhen Bao'an Traditional Chinese Medicine Hospital Group, Shenzhen, China; 5 Hospital Infection Department, Guangdong Provincial Hospital of Traditional Chinese Medicine, Shenzhen, China

**Keywords:** continuing care, family care, new media, rehabilitation, self-management, stroke

## Abstract

**Introduction:**

Persistent functional impairment and psychological distress are common after stroke, highlighting the need for effective post-discharge nursing strategies. We performed a retrospective cohort study evaluating the associations of a family-centered, new-media continuous nursing intervention on stroke recovery outcomes.

**Methods:**

The study included 107 patients with first-ever ischemic stroke who received either routine post-discharge care or a family-centered new-media continuous nursing intervention. Functional status, depressive symptoms, and quality of life were assessed at baseline and 6 months. Rehabilitation adherence, platform engagement indicators, and selected serum biomarkers related to neuroplasticity and inflammation were analyzed. Multivariable models were used to adjust for baseline clinical factors.

**Results:**

At 6 months, the intervention group showed significantly greater improvements in Barthel Index scores, larger reductions in Patient Health Questionnaire-9 scores, and greater gains in quality of life compared with routine care. Rehabilitation compliance and medication adherence were higher in the intervention group. Within this group, greater platform engagement was associated with larger improvements in depressive symptoms and quality of life. In addition, patients receiving the intervention exhibited greater increases in serum brain-derived neurotrophic factor and endothelial progenitor cell counts, along with more pronounced reductions in IL-6 and TNF-α. Participation in the intervention remained independently associated with functional and psychological improvement after adjustment.

**Discussion:**

Family-centered new-media continuous nursing is associated with improved functional independence, psychological recovery, adherence behaviors, and favorable biological changes in patients with ischemic stroke.

## Introduction

Stroke is a leading cause of long-term disability and mortality worldwide, particularly among middle-aged and older adult(s) individuals (1, 2). Among stroke subtypes, ischemic stroke—caused by sudden reduction or cessation of cerebral blood flow—accounts for the majority of cases, and its management has been guided by the Chinese Medical Association's primary–care guidelines on diagnosis and treatment (3). Despite major advances in acute care, including thrombolysis and mechanical thrombectomy, many survivors experience persistent deficits in motor function, language, cognition and emotional regulation that substantially impair activities of daily living (ADL) and quality of life (4). It is estimated that roughly 75% of stroke survivors endure residual disability severe enough to limit self-care and independence, thereby imposing heavy burdens on family caregivers and the healthcare system (2).

Traditional transitional care models, often fail to provide timely, individualized support during the critical post-discharge period (5). Such gaps highlight the urgent need for structured, comprehensive transitional care that equips both patients and caregivers with the skills, knowledge and confidence to manage recovery at home.

Over the past decade, new-media technologies, including mobile applications, social media platforms and web-based education modules, have revolutionized possibilities for continuous, interactive healthcare delivery (6, 7). These platforms may facilitate real-time communication between patients, caregivers and nursing staff; deliver multimedia health education, automate reminders for medication and exercises, and foster peer support networks. In other chronic disease contexts, such as diabetes and heart failure, higher “dose” of engagement, measured by login frequency, session duration and content viewed, has been positively correlated with clinical improvements (8). Regarding stroke rehabilitation, studies incorporating tele-nursing or web-based interventions have demonstrated improved functional recovery and patient satisfaction as well: tele-based rehabilitation and structured remote monitoring can achieve outcomes comparable to conventional care while improving continuity and accessibility of long-term management in patients with heart failure or stroke (9, 10). Yet, in stroke care, most tele-nursing studies remain narrowly focused on physical recovery metrics (e.g., NIHSS and Barthel Index scores) without systematically assessing psychosocial outcomes (such as depression), quantifying objective engagement behaviors, or capturing user satisfaction and perceived barriers.

Few digital nursing programs incorporate validated mood assessments, such as the Patient Health Questionnaire-9 (PHQ-9), in combination with functional and quality-of-life measures. Furthermore, the relationship between platform engagement metrics and improvements in mood or ADL has not been rigorously examined, and qualitative feedback on user satisfaction, barriers to use and suggestions for improvement remains under-reported, limiting our understanding of real-world feasibility and acceptability.

To address these issues, we analyzed the treatment outcomes in a cohort study involving 107 ischemic stroke patients (52 handled by routine-care and 55 handled by family-centered new-media continuous nursing intervention) discharged from our hospital between January 2022 and December 2023. The intervention leveraged a purpose-built stroke home-care platform integrating offline motivational interviewing with online educational modules (text, images and video) and behavioral skills demonstrations (11). From a theoretical point, the intervention of the family-centered new-media continuous nursing is made up by two catergories, which are self-management theory and social support theory, both of which are widely applied in chronic disease care. Self-management theory emphasizes individuals' active role in managing symptoms, treatment regimens, lifestyle adaptations, and psychosocial challenges through skills such as goal setting, self-monitoring, and problem solving (12). Digital health platforms are particularly well suited to operationalize these principles by delivering structured education, reinforcing self-care behaviors, and enabling timely feedback (13). Complementarily, social support theory highlights the role of emotional, informational, and instrumental support from family members and healthcare providers in buffering stress and facilitating recovery (14). In the context of stroke rehabilitation, caregiver involvement and continuous nurse–patient communication may enhance adherence, psychological adjustment, and engagement with rehabilitation activities. Together, these frameworks provide a conceptual basis for hypothesizing that a family-centered, technology-assisted nursing model can improve functional and psychological outcomes after stroke.

Our primary objectives were to compare changes in functional independence (ADL scores), depressive symptoms (PHQ-9) and health-related quality of life (SF-36) between groups. Secondary objectives included quantifying platform engagement metrics, examining their correlations with clinical outcomes, and identifying independent predictors of rehabilitation success through multivariable regression, adjusting for baseline NIHSS, age and caregiver relationship. Beyond clinical effectiveness, technology-assisted continuous care interventions may offer important implementation-related advantages, such as reducing the burden on in-person healthcare resources, optimizing nursing workforce allocation, and improving continuity of care following hospital discharge. At the same time, implementation challenges, including variability in digital literacy, access to technology, and the need for standardized care pathways, remain important considerations. Therefore, evaluating interventions within this broader implementation context is essential to inform their real-world applicability and potential impact on healthcare systems.

We hypothesized that, compared with routine care, the new-media intervention would yield greater improvements in ADL, mood and quality of life, demonstrating a positive dose–response relationship between engagement and outcomes. By integrating multimodal outcome measures and detailed process data within a behavioral-theory framework that emphasizes the importance of individuals managing their own health and making decisions regarding their care that is facilitated by the new-media platform as well as highlighting the role of caregivers and the importance of social networks in promoting recovery, this study aims to elucidate not only the efficacy but also the mechanisms by which digital continuous nursing promotes post-stroke recovery.

## Materials and methods

### Study design and participants

**Figure 1:**
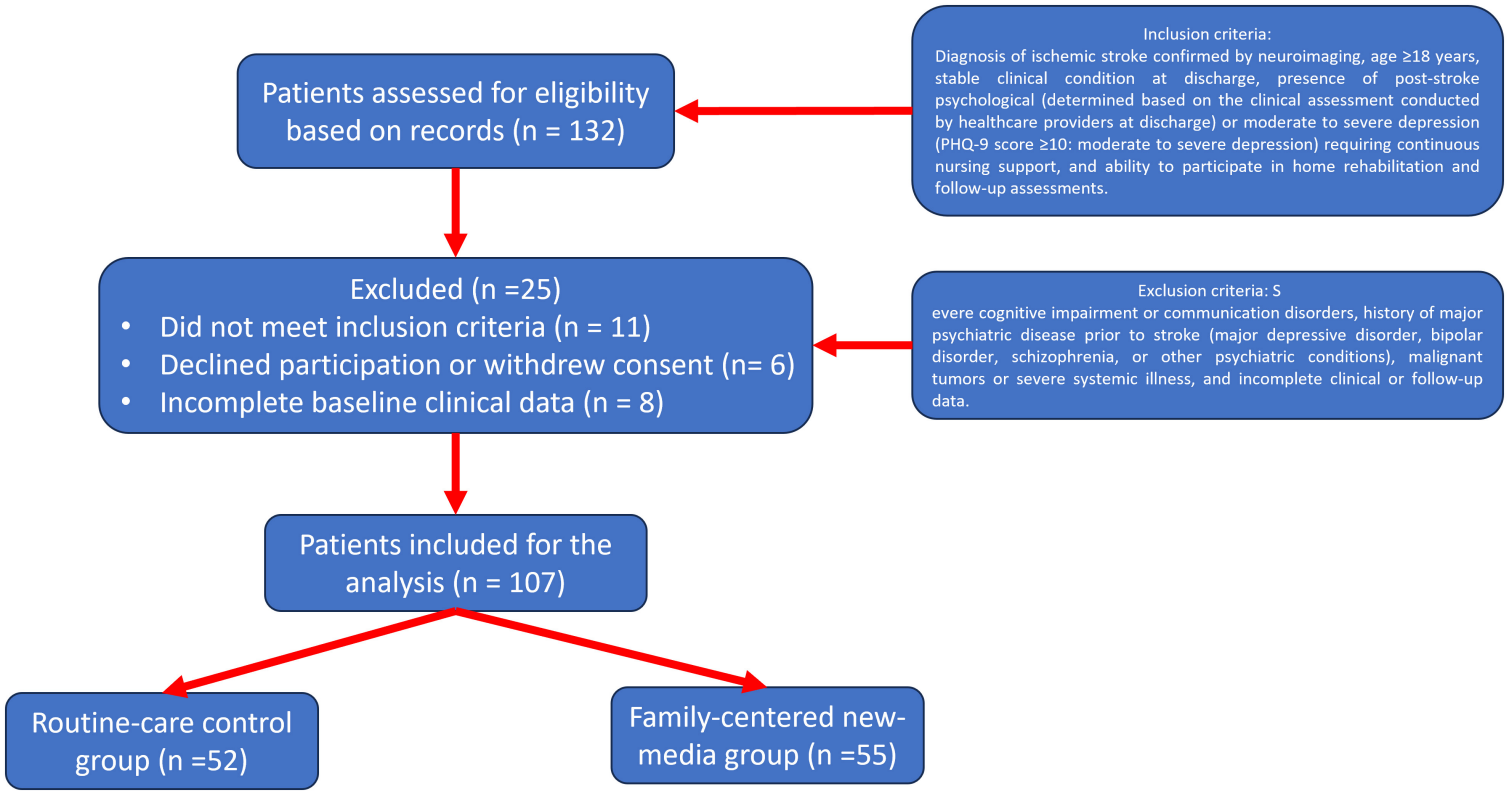
Flowchart of patient selection.

This study was a retrospective controlled cohort study conducted at the Guangdong Provincial Hospital of Traditional Chinese Medicine. Patients with first-ever ischemic stroke who were hospitalized and subsequently entered the post-discharge continuing-care phase between January 2022 and December 2023 were screened. Inclusion criteria were diagnosis of ischemic stroke confirmed by neuroimaging, age ≥18 years, stable clinical condition at discharge, presence of post-stroke psychological (determined based on the clinical assessment conducted by healthcare providers at discharge) or moderate to severe depression (PHQ-9 score ≥10: moderate to severe depression) requiring continuous nursing support, and ability to participate in home rehabilitation and follow-up assessments. Exclusion criteria included severe cognitive impairment or communication disorders, history of major psychiatric disease prior to stroke (major depressive disorder, bipolar disorder, schizophrenia, or other psychiatric conditions), malignant tumors or severe systemic illness, and incomplete clinical or follow-up data. While randomization was not feasible due to the retrospective nature of the study, patients in the family-centered new-media group were those who opted for this additional intervention, either through their own preference or based on the recommendation of healthcare providers. The routine-care group received standard post-discharge care. Eligible patients were allocated according to the type of post-discharge continuing care received into a routine-care control group (*n* = 52) and a family-centered new-media continuous nursing group (*n* = 55). The inclusion and exolcusion of the patients was shown in Figure 1. The study protocol was approved by the institutional ethics committee, and all participants had provided written informed consent for the use of anonymized clinical data.

### Family-centered new-media continuous nursing intervention

Patients in the family-centered new-media continuous nursing group received a structured continuing-care program delivered through a new-media platform after discharge. The intervention integrated home-based rehabilitation guidance, health education related to stroke secondary prevention and daily self-management, psychological support, and interactive communication between patients, caregivers, and nursing staff: briefly, the psychological support component involved regular online counseling and motivational interviewing, facilitated through the platform, and aimed at helping patients and their caregivers manage emotional challenges related to stroke recovery. This support also included mental health resources and strategies for managing stress, anxiety, and depressive symptoms. Family members actively participated in supervising rehabilitation exercises, assisting with daily care activities, and providing emotional support, forming a patient–family–nurse collaborative care model. Nursing staff monitored patient progress through the platform, provided individualized feedback, and responded to patient or caregiver inquiries within a standardized time frame to ensure continuity of care throughout the follow-up period, and followed the intervention guidelines, which included specific instructions on providing feedback, offering support, and addressing patient concerns in a structured manner. All the nurses involved in the intervention were uniformly trained in the standardized protocol, including the use of the new-media platform, communication techniques, and how to provide rehabilitation guidance and psychological support. Patients in the routine-care control group received standard post-discharge nursing care, including discharge education, routine outpatient follow-up, and telephone guidance when necessary, without structured new-media or family-centered continuous nursing support. For the current study, patients were expected to log into the platform at least twice per week and complete at least 50% of the assigned rehabilitation modules to be considered engaged with the intervention.

### Baseline clinical assessment

Baseline demographic and clinical data were extracted from electronic medical records, including age, sex, stroke subtype, vascular risk factors, and caregiver relationship. Neurological impairment at discharge was assessed using the National Institutes of Health Stroke Scale, and functional independence was evaluated using the Barthel Index (BI). Cognitive status was screened at discharge according to routine clinical practice to ensure patients were capable of participating in home-based rehabilitation and completing follow-up assessments. Baseline psychological status and quality of life (SF-36: a widely recognized and validated instrument for measuring various dimensions of QoL in stroke patients), where recorded in routine nursing or rehabilitation evaluations, were also extracted to support longitudinal comparisons.

### Outcome measures

Psychological status, self-management ability, functional recovery, and quality of life were assessed at baseline and at 6-month follow-up using validated instruments routinely applied in clinical nursing practice. Functional recovery was measured using the BI scale. Depressive symptoms were assessed using the Patient Health Questionnaire-9, which was routinely administered as part of post-stroke psychological screening, and clinically meaningful improvement was evaluated based on changes in total score. Health-related quality of life was assessed using a standardized quality-of-life instrument documented in the medical records, and changes in overall and domain-specific scores were analyzed as secondary outcomes. Rehabilitation compliance was recorded through the platform logs, which tracked the completion rates of prescribed rehabilitation modules and exercises. Engagement-related indicators, including platform login frequency and rehabilitation module completion rate, were collected as exploratory measures to characterize patterns of platform use and were not pre-specified as primary or secondary study outcomes. These data were used to assess the consistency and engagement with rehabilitation activities over the 6-month follow-up period, and compliance was evaluated based on follow-up records, reflecting patients' continuity, frequency, and completion of prescribed home-based rehabilitation activities during the recovery period.

### New-media engagement, caregiver participation, and adherence indicators

For patients in the family-centered new-media continuous nursing group, platform operation logs and follow-up documentation were retrospectively extracted to obtain objective indicators of engagement and caregiver participation. These indicators included frequency and continuity of platform use, completion of rehabilitation or education content, documented interactions between patients, caregivers, and nursing staff, and caregiver involvement in daily rehabilitation supervision. Medication adherence was assessed based on documented reports during follow-up visits, where patients were asked about their medication intake and whether it adhered to the prescribed regimen. These variables were analyzed as derived measures to explore their associations with functional, psychological, and quality-of-life outcomes, without redefining intervention exposure or group allocation.

### Laboratory and biological measurements

Peripheral venous blood samples collected from all the patients according to routine clinical protocols at baseline and at the 6-month follow-up were analyzed for biological indicators related to neuroplasticity, inflammation, and vascular repair. Serum brain-derived neurotrophic factor (BDNF) level was measured using enzyme-linked immunosorbent assay, inflammatory status was evaluated by measuring IL-6 and TNF-α concentrations, and endothelial progenitor cell (EPC) counts were determined by flow cytometry. EPCs play a key role in vascular repair and angiogenesis, both of which are important for stroke recovery. Where available from routine laboratory testing, additional nonspecific inflammatory indicators, such as high-sensitivity C-reactive protein or neutrophil-to-lymphocyte ratio, were extracted as supplementary markers to support interpretation of systemic inflammatory status and were treated as exploratory outcomes.

### Statistical analysis

Statistical analyses were performed using SPSS software. Continuous variables were expressed as mean ± standard deviation, and categorical variables were expressed as frequencies and percentages. Engagement-related variables, including platform login frequency and rehabilitation module completion rate, were modeled as continuous predictors in that they reflect the intensity of patient participation over time and enable assessment of potential dose–response relationships between engagement level and clinical outcomes. Between-group comparisons were conducted using independent-sample *t* tests or χ^2^ tests as appropriate. Longitudinal changes in outcomes were analyzed using repeated-measures or mixed-effects models incorporating group, time, and group-by-time interaction terms, with adjustment for baseline age, neurological impairment, functional status, and other clinically relevant covariates. Multivariable regression analyses were used to examine associations between new-media engagement intensity, caregiver participation, biological indicators, and patient outcomes. Effect sizes and 95% confidence intervals were reported where applicable, and a two-sided *P* value < 0.05 was considered statistically significant.

## Results

### Baseline characteristics

A total of 107 patients were included in the final analysis, comprising 52 patients in the routine-care control group and 55 patients in the family-centered new-media continuous nursing group (Figure 1). Baseline demographic and clinical characteristics are summarized in Table 1. There were no statistically significant differences between the two groups with respect to age, sex distribution, vascular risk factors, stroke severity as assessed by NIHSS at discharge, baseline BI score, baseline depressive symptom severity measured by PHQ-9, or baseline quality-of-life scores (all *P* > 0.05), indicating good baseline comparability.

**Table 1:** Baseline demographic and clinical characteristics.

**Variable**	**Routine-care control group (*n* = 52)**	**Family-centered new-media group (*n* = 55)**	**SMD**	***P* value**
Age (years)	63.1 ± 9.4	62.4 ± 9.7	0.07	0.69
Male, *n* (%)	31 (59.6)	34 (61.8)	–	0.814
Hypertension, *n* (%)	33 (63.5)	36 (65.5)	–	0.83
Diabetes mellitus, *n* (%)	15 (28.8)	16 (29.1)	–	0.973
Dyslipidemia, *n* (%)	19 (36.5)	21 (38.2)	–	0.856
Smoking history, *n* (%)	18 (34.6)	17 (30.9)	–	0.683
NIHSS score at discharge	6.2 ± 2.4	6.0 ± 2.3	0.08	0.65
Barthel index at baseline	55.8 ± 12.1	56.6 ± 12.6	0.06	0.738
PHQ-9 score at baseline	12.4 ± 3.1	12.2 ± 3.2	0.06	0.736
Quality-of-life score at baseline	49.3 ± 10.0	49.8 ± 9.8	0.05	0.796

SMD, standardized mean differences.

An SMD < 0.10 indicates negligible baseline imbalance.

### Changes in depressive symptoms and quality of life

At the 6-month follow-up, both groups demonstrated significant improvement in depressive symptoms and quality of life compared with baseline; however, the magnitude of improvement was significantly greater in the family-centered new-media continuous nursing group (Table 2).

**Table 2:** Changes in depressive symptoms and quality of life at 6-month follow-up.

**Outcome**	**Group**	**Baseline**	**6 months**	**Change (Δ)**	**SMD**	***P* (between-group Δ)**
PHQ-9 score	Routine-care	12.4 ± 3.1	9.1 ± 3.2	−3.3 ± 2.6	0.99	**< 0.001**
	New-media	12.2 ± 3.2	6.2 ± 2.7	−6.0 ± 2.8		
Quality-of-life score	Routine-care	49.3 ± 10.0	57.2 ± 9.6	+7.9 ± 7.4	1.03	**< 0.001**
	New-media	49.8 ± 9.8	65.4 ± 8.7	+15.6 ± 7.6		

SMD, standardized mean differences.

An SMD < 0.10 indicates negligible baseline imbalance.

The bold values indicates *P* < 0.05.

PHQ-9 scores decreased from in the new-media group, compared with a reduction from in the routine-care group, resulting in a significantly larger between-group difference in score change (*P* < 0.001). Similarly, SF36 scores increased more markedly in the new-media group than in the routine-care group, with a significant between-group difference in improvement (*P* < 0.001). In addition to statistical significance, SMD demonstrated large effect sizes for improvements in depressive symptoms and QoL in the family-centered new-media group compared with routine care (Table 2).

**Figure 2:**
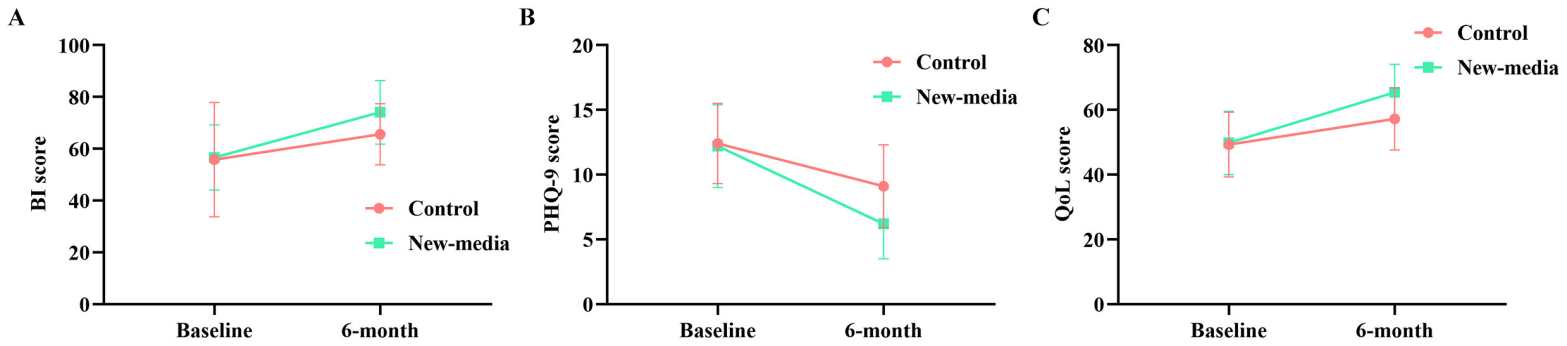
Longitudinal changes in functional, psychological, and quality-of-life outcomes. **(A)** Changes in Barthel Index (BI) scores from baseline to 6-month follow-up. **(B)** Changes in depressive symptoms assessed by the Patient Health Questionnaire-9 (PHQ-9) from baseline to 6 months. **(C)** Changes in health-related quality-of-life (QoL) scores from baseline to 6-month follow-up in the two groups.

The longitudinal trajectories of BI, PHQ-9, and quality-of-life scores are illustrated in Figures 2A–C, which visually demonstrate a greater rate and extent of improvement in the new-media group over the 6-month follow-up period.

### New-Media engagement and caregiver participation

Among patients receiving family-centered new-media continuous nursing, platform engagement and careygiver participation were consistently observed during follow-up (Table 3). Patients logged into the platform an average of 18.6 ± 7.9 times per month, with a mean rehabilitation module completion rate of 76.4% ± 15.2%. Interactive communication with nursing staff occurred frequently, and 83.6% of patients maintained continuous platform use for at least 3 months. Active caregiver participation was documented in 80.0% of cases, supporting effective implementation of the family-centered care model.

**Table 3:** New-media platform engagement and caregiver participation indicators.

**Indicator**	**Mean ±SD**
Platform logins (times/month)	18.6 ± 7.9
Rehabilitation module completion rate (%)	76.4 ± 15.2
Interactive communications (times/month)	6.8 ± 3.1
Continuous platform use ≥3 months, *n* (%)	46 (83.6)
Active caregiver participation documented, *n* (%)	44 (80.0)

### Rehabilitation compliance and adherence

Rehabilitation compliance and treatment adherence outcomes are shown in Table 4. The proportion of patients achieving high rehabilitation compliance was significantly higher in the new-media group than in the routine-care group (*P* = 0.005), while the proportion of patients with low compliance was markedly lower. In addition, documented medication adherence was more frequent in the new-media group compared with the routine-care group (*P* = 0.039), indicating improved behavioral adherence associated with continuous nursing support.

**Table 4:** Rehabilitation compliance and adherence outcomes.

**Variable**	**Routine-care control (*n* = 52)**	**Family-centered new-media (*n* = 55)**	***P* value**
High rehabilitation compliance, *n* (%)	20 (38.5)	36 (65.5)	**< 0.001**
Moderate compliance, *n* (%)	18 (34.6)	15 (27.3)	**< 0.001**
Low compliance, *n* (%)	14 (26.9)	4 (7.3)	**< 0.001**
Documented medication adherence, *n* (%)	31 (59.6)	43 (78.2)	**< 0.001**

The bold values indicates *P* < 0.05.

### Changes in biological markers

Changes in biological markers related to neuroplasticity, inflammation, and vascular repair are summarized in Table 5. At follow-up, patients in the family-centered new-media group exhibited significantly greater increases in serum BDNF levels and EPC counts compared with the routine-care group (both *P* < 0.001). Concurrently, inflammatory markers including IL-6 and TNF-α showed more pronounced reductions in the new-media group than in the routine-care group (both *P* < 0.001).

**Table 5:** Changes in biological markers from baseline to 6-month follow-up.

**Marker**	**Group**	**Baseline**	**6 months**	**Change (Δ)**	**SMD**	***P* (between-group Δ)**
BDNF (pg/mL)	Routine-care	11.2 ± 3.0	13.4 ± 3.1	+2.2 ± 2.2	1.25	**< 0.001**
	New-media	11.1 ± 3.1	16.3 ± 3.2	+5.2 ± 2.5		
IL-6 (pg/mL)	Routine-care	7.6 ± 2.1	6.3 ± 1.9	−1.3 ± 1.5	0.96	**< 0.001**
	New-media	7.5 ± 2.2	4.7 ± 1.6	−2.8 ± 1.6		
TNF-α (pg/mL)	Routine-care	9.4 ± 2.4	8.3 ± 2.2	−1.1 ± 1.6	0.79	**< 0.001**
	New-media	9.3 ± 2.5	6.9 ± 2.0	−2.4 ± 1.7		
EPC count (cells/μL)	Routine-care	43.2 ± 12.0	49.1 ± 12.4	+5.9 ± 7.8	1.18	**< 0.001**
	New-media	42.6 ± 12.3	58.4 ± 13.0	+15.8 ± 9.1		

SMD, standardized mean differences.

An SMD < 0.10 indicates negligible baseline imbalance.

The bold values indicates *P* < 0.05.

Effect size analysis further indicated moderate-to-large standardized mean differences for changes in BDNF, inflammatory cytokines, and EPC counts (Table 5). These findings suggest that continuous family-centered nursing may be associated with favorable biological changes that parallel improvements in psychological and functional outcomes.

### Association between new-media engagement and outcomes

**Figure 3:**
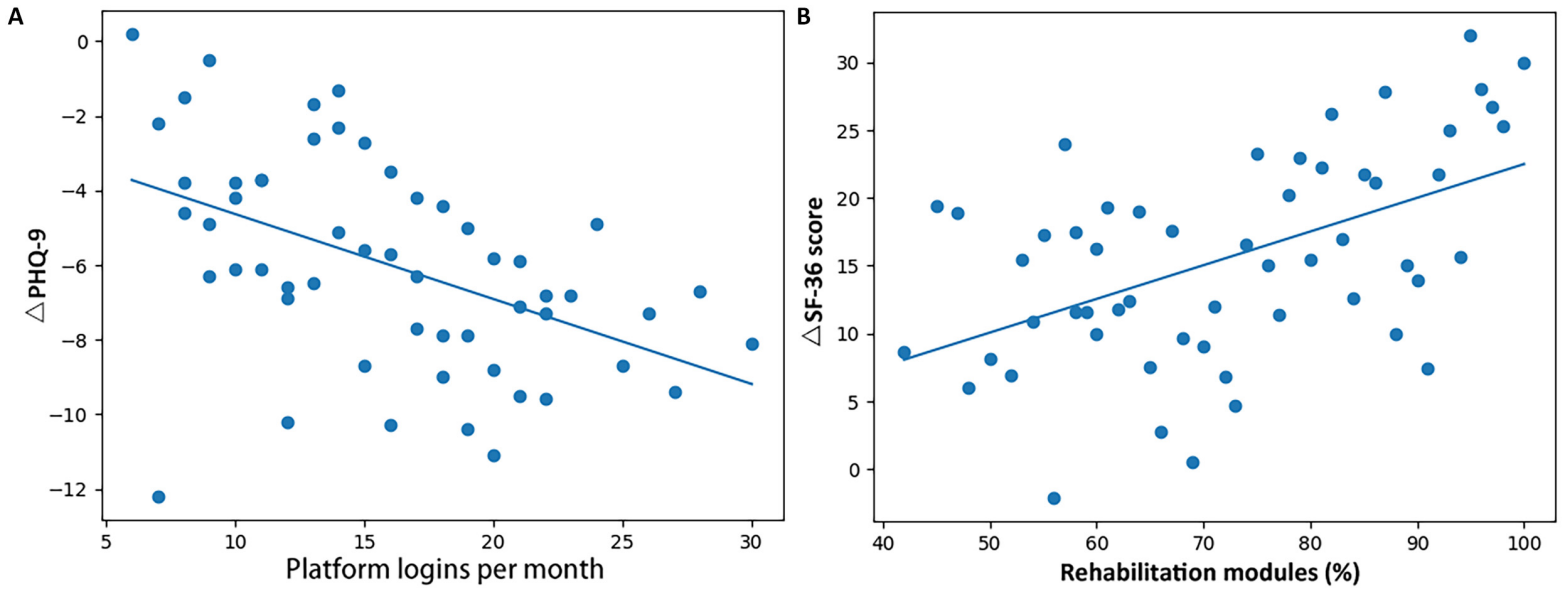
Associations between new-media engagement and clinical outcomes in the family-centered new-media group (*n* = 55). **(A)** Scatter plot showing the association between average monthly new-media platform login frequency and change in PHQ-9 score over 6 months. **(B)** Scatter plot illustrating the relationship between rehabilitation module completion rate and change in QoL over 6 months.

Correlation analyses demonstrated significant associations between the intensity of new-media engagement and clinical outcomes within the family-centered new-media group. As shown in Figure 3A, greater platform login frequency was significantly associated with larger reductions in PHQ-9 scores (*P* = 0.001). Similarly, higher rehabilitation module completion rates were positively correlated with improvements in quality-of-life scores (Figure 3B) (*P* < 0.001).

These findings indicate a dose–response relationship between engagement with the new-media platform and improvements in psychological wellbeing and quality of life.

### Multivariable analysis of functional and psychological outcomes

Results of the multivariable mixed-effects models are presented in Table 6. After adjustment for age, baseline NIHSS score, baseline BI scale, baseline PHQ-9 score, and comorbidities, the group × time interaction remained a significant independent predictor of both functional recovery and depressive symptom improvement. Specifically, participation in family-centered new-media continuous nursing was independently associated with greater improvement in BI scores (*P* < 0.001) and greater reduction in PHQ-9 scores (*P* < 0.001).

**Table 6:** Multivariable mixed-effects model for functional and psychological outcomes.

**Outcome**	**Predictor**	**β (95% CI)**	***P* value**
Barthel Index	Group × Time	**+9.8 (6.1 to 13.5)**	**< 0.001**
	Baseline NIHSS (per 1 point)	−2.1 (−3.2 to −1.0)	**< 0.001**
	Age (per 1 year)	−0.2 (−0.4 to 0.0)	0.061
PHQ-9	Group × Time	**−2.7 (−3.6 to** **−1.8)**	**< 0.001**
	Baseline PHQ-9 (per 1 point)	+0.5 (0.3 to 0.7)	**< 0.001**
	Baseline NIHSS (per 1 point)	+0.3 (0.1 to 0.6)	0.012

The bold values indicates *P* < 0.05.

## Discussion

The present study demonstrates that an association between family-centered, new-media continuous nursing intervention and post-stroke recovery across functional, psychological, and biological domains, compared with routine follow-up care alone. Patients in the intervention group showed significantly greater improvements in activities of daily living, depressive symptoms, and quality of life over the 6-month follow-up period. Specifically, the adjusted group × time effect indicated an independent gain of approximately 9.8 points in the BI scale and a reduction of 2.7 points in PHQ-9 scores attributable to the intervention, even after controlling for baseline NIHSS score, age, and baseline psychological status. For the BI scale, a change of three points is generally considered to represent a clinically meaningful improvement in functional independence, while for the PHQ-9, a reduction of three points is typically regarded as clinically significant in terms of depressive symptom reduction. These thresholds further emphasize the relevance of the observed improvements in the intervention group, where BI scores increased by approximately 9.8 points and PHQ-9 scores decreased by 2.7 points. These changes are not only statistically significant but also exceed the minimal clinically important difference, further supporting the potential of family-centered new-media continuous nursing to foster meaningful recovery outcomes in stroke patients.

An important finding of this study is the improvement in depressive symptoms observed during post-stroke recovery, as assessed using the (PHQ-9. Post-stroke depression is widely recognized as a major determinant of functional prognosis and long-term disability (11, 15). In the current cohort, although both groups experienced alleviation of depressive symptoms, the magnitude of PHQ-9 reduction was markedly greater in the family-centered new-media group (-6.0 vs. −3.3) (15). This improvement was accompanied by parallel gains in quality-of-life scores, suggesting that psychological recovery and perceived wellbeing progressed in tandem. The longitudinal trajectories further demonstrate a steeper and more sustained improvement in the intervention group across functional outcomes and depression-related measures (4, 16).

Importantly, the curent study extends existing tele-nursing literature by quantitatively linking intervention engagement to clinical outcomes (6, 8, 17). Objective platform-use metrics revealed a clear dose–response relationship, with higher login frequency and rehabilitation module completion rates significantly correlated with greater reductions in PHQ-9 scores and larger gains in QoL measures (8). These associations, indicate that continuous, interactive exposure to structured nursing guidance, rather than mere access to digital tools, is critical for achieving meaningful recovery benefits (18). Such findings support a mechanistic role of sustained engagement in mediating behavioral adherence and psychological adaptation (19).

Nevertheless, the primary outcomes of this study, including functional recovery (measured by the BI scale) and depressive symptom reduction (measured by the PHQ-9 score), were pre-specified and thus provide more robust evidence of the effectiveness of the intervention. These findings still require further validation in prospective trials to confirm their generalizability. In contrast, the exploratory outcomes, including the engagement metrics and biological markers, are more hypothesis-generating in nature. These findings suggest interesting potential mechanisms through which the intervention might work but should not be interpreted as definitive proof of causality. Future studies with a more rigorous design, such as randomized controlled trials, will be needed to confirm these exploratory findings and to establish the underlying mechanisms in a more definitive manner.

Rehabilitation compliance represents a potential pathway through which the intervention may be associated with observed outcomes. In this cohort, the proportion of patients achieving high rehabilitation compliance was significantly higher in the new-media group compared with routine care, while the proportion with low compliance was substantially reduced (16). Improved medication adherence further supports the role of family participation and real-time nursing supervision in reinforcing health behaviors (2, 5). These behavioral improvements provide a plausible explanation for the superior functional outcomes observed, particularly given the established association between adherence and post-stroke neuroplastic recovery (4).

At the biological level, the study provides evidence that family-centered continuous nursing is associated with favorable neuroimmune modulation. Patients in the intervention group exhibited significantly greater increases in serum BDNF and EPC counts, alongside more pronounced reductions in IL-6 and TNF-α, compared with controls (20). These biomarkers are closely linked to neuroplasticity, angiogenesis, and inflammatory regulation (21, 22). The parallel changes observed across biological, functional, and psychological domains support the hypothesis that structured rehabilitation and psychosocial support may be linked to a more permissive internal environment for neural repair (20–22). While the changes in biomarkers were observed in the intervention group, the exact mechanisms through which the intervention influenced these markers remain unclear (21, 22).

The multivariable mixed-effects analyses further clarify determinants of recovery. Beyond the intervention, baseline stroke severity (NIHSS) and baseline psychological burden remained independently associated with outcomes (11, 15). Age showed only a marginal association with functional recovery and did not significantly attenuate the intervention effect, suggesting that the benefits of family-centered new-media nursing may extend across a broad age range (1). This observation has important implications for scalability in aging populations with a high burden of stroke-related disability (2). Although caregiver-related outcomes were not directly measured in this study, findings outlined in the current study suggests that the involvement in home-based and remotely delivered interventions may place additional demands on caregivers, potentially increasing caregiving burden, stress, and role strain. Remote and technology-assisted interventions may not always adequately address caregivers' own needs, and expectations for caregiver participation may inadvertently shift responsibilities from healthcare providers to family members. At the same time, caregiver engagement may also confer benefits, including enhanced understanding of stroke recovery processes, improved communication with healthcare teams, and greater emotional support for patients. These dual considerations highlight the importance of balancing patient benefits with caregiver wellbeing, and future studies should explicitly evaluate the effects of such interventions on caregivers' health, burden, and support needs.

From an implementation perspective, the findings highlighted in the current study suggest that the evaluated intervention may have implications beyond individual patient outcomes. By supporting continuous care, this approach may help alleviate pressure on outpatient services and reduce reliance on resource-intensive face-to-face follow-up. In nursing practice, technology-assisted interventions may facilitate more efficient use of human resources by enabling targeted, needs-based follow-up rather than uniform in-person care. However, successful implementation depends on factors such as patient engagement, digital accessibility, and integration within existing care workflows. Addressing these considerations is critical to maximizing the potential system-level impact of such interventions and supports the need for future studies focusing on cost-effectiveness, scalability, and implementation feasibility.

Thus, several limitations merit consideration. First, the retrospective, non-randomized, and single-center design introduces potential selection bias and limit the generalizability of the findings to other healthcare settings or populations. Second, as patients opting for the new-media intervention may have differed in motivation or digital literacy, it is possible that patients who experienced improvements in their functional and psychological outcomes or have higher digital literacy may have been more motivated to engage with the platform, leading to further improvements. While our study design does not allow us to definitively determine the direction of this relationship, we acknowledge this as a limitation and suggest that future prospective studies explore the temporal relationship between engagement and outcomes more rigorously (6). Baseline comparability across demographic, clinical, functional, and psychological variables reduces, but does not eliminate this concern (16). Future studies explore how these variables can be controlled or adjusted for. Third, engagement metrics derived from platform logs may not fully capture qualitative aspects of interaction or caregiver competence (23). Fourth, although biological markers were systematically analyzed, they were limited to peripheral blood indices and cannot directly reflect central nervous system processes (20–22, 24, 25). Additionaly, the 6-month follow-up period, while clinically relevant, does not address the durability of benefits or long-term outcomes such as recurrent stroke or rehospitalization. Finally, the study only assessed psychological recovery using the PHQ-9, which focuses specifically on depression and does not capture other important dimensions of post-stroke psychological recovery, such as anxiety, emotional adjustment, coping capacity, or post-traumatic growth. Therefore, the findings related to psychological outcomes should be interpreted as reflecting changes in depressive symptomatology rather than comprehensive psychological recovery, and the observational and retrospective nature of the current study demonstrated that causality cannot be inferred from the findings.

In summary, the study shows that a family-centered new-media continuous nursing model was associated with multidimensional improvements for stroke survivors, encompassing functional independence, psychological recovery, behavioral adherence, and favorable biological changes. The alignment between clinical outcomes, engagement indicators, and biomarker trends underscores the internal coherence of the current findings. These results support the integration of structured digital nursing platforms into post-stroke transitional care and provide a solid empirical basis for future prospective and randomized investigations. However, given the findings of the current study relied on the retrospective design, future research should focus on randomized controlled trials to confirm long-term benefits and cost-effectiveness of family-centered, new-media continuous nursing interventions before they can be considered for widespread policy adoption.

## Data Availability

The raw data supporting the conclusions of this article will be made available by the authors, without undue reservation.
